# Production of a Natural Antibody to the Mouse Polyoma Virus Is a Multigenic Trait

**DOI:** 10.1534/g3.111.001701

**Published:** 2012-03-01

**Authors:** Erik Andrews, Palanivel Velupillai, Chang Kyoo Sung, David R. Beier, Thomas Benjamin

**Affiliations:** * Department of Microbiology and Immunobiology; † Department of Genetics, Harvard Medical School, Boston, Massachusetts 02115

**Keywords:** natural antibody, polyoma virus, multigenic inheritance, epistasis

## Abstract

MA/MyJ mice express a natural antibody to the highly oncogenic polyoma virus. C57BR/cdJ mice lack this antibody but mount an adaptive T-cell response to the virus. Analysis of F2 progeny of a cross between these strains reveals a pattern of inheritance of expression of the natural antibody involving two genes in an epistatic relationship.

Two forms of resistance in inbred strains of mice to the oncogenic effects of polyoma virus emerged in the course of studies of the effects of ionizing radiation on development of virus-induced tumors (Carroll *et al.* 1999). C57BR/cdJ mice (BR) are resistant to tumor induction on the basis of a well characterized cytolytic T-cell response targeting the major virus transforming protein (Lukacher and Wilson 1998). This adaptive immune response is readily overcome by an immunosuppressive dose of radiation (Carroll *et al.* 1999; Lukacher *et al.* 1995). In contrast, MA/MyJ mice (MA) maintain their resistance to tumor induction by the virus after irradiation. MA also manifest another unusual feature, which is resistance to infection by a highly virulent strain of polyoma virus that rapidly kills newborn BR mice and mice of other strains. This resistance is transmitted as a dominant or semidominant trait (Carroll *et al.* 1999).

Sera from normal MA mice show high levels of a natural antibody capable of neutralizing the virus. A hemagglutination-inhibition (HA-I) assay (Carroll *et al.* 2007) based on serial twofold dilutions of sera reveals the presence of this natural antibody. Titers are in the range of 2560 to 5120 for MA, 40 to 80 for BR, and 320 to 640 for [MAxBR]F1 animals. The neutralizing activity of sera from uninfected MA is roughly an order of magnitude lower than that found in experimentally or naturally infected mice (Carroll *et al.* 2007). It nevertheless accounts in all likelihood for all or part of the unusual resistance of MA. Here, we report the use of genome-wide single nucleotide polymorphism (SNP) analysis of the F2 progeny of a cross between MA and BR to identify the genetic loci controlling production of the natural antibody.

MA and BR were crossed in both parental directions. F1 mice from each pairing were used to generate a total of 192 F2 animals for phenotyping (Figure 1). No difference based on parental pairings of the F1s was apparent. HA-I titers fell almost exclusively into three discrete ranges corresponding to those found for parental and F1 mice: 2560 to 5120, high titer (MA-like); 320 to 640, intermediate (F1-like); and 40 to 80, low titer (BR-like). Adding the three outliers with titers of 1280 and 10,240 to the high titer group produces a distribution of 50 low titer, 93 intermediate, and 49 high titer, which is consistent with a monogenetic pattern of inheritance (χ^2^ test of difference = 0.1979, *P* = 0.91).

**Figure 1:**
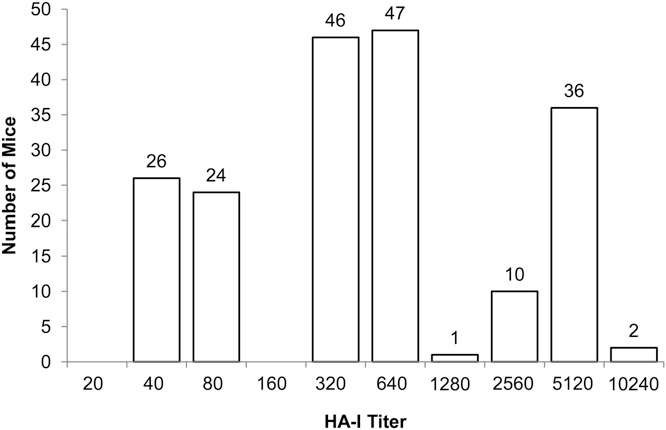
Results of a hemagglutination inhibition assay on F2 mice. Serial twofold dilutions of sera from normal 4- to 5-week-old mice were mixed with an equal volume of virus suspension and incubated at room temperature for 45 min. An equal volume of guinea pig red blood cell suspension was added, and cells were allowed to settle for 4 hours at 4°C. HA-I titers are expressed as the greatest dilution of sera that prevents agglutination of the red blood cells by the virus (Carroll *et al.* 2007).

To identify the genetic map position of the locus or loci controlling antibody levels, DNAs from 30 high-titer and 30 low-titer F2 mice were analyzed. A genome-wide SNP array was used with 1449 SNPs, of which 686 were informative between MA and BR (Figure 2). Quantitative trait loci (QTL) analysis across the genome revealed two dominant peaks (Figure 2A), one on chromosome 4 [logarithm of the odds (LOD) 18.06, *P* < 0.00001] and the other on chromosome 7 (LOD 18.06, *P* < 0.00001). The next greatest peak, on chromosome 19 (LOD 3.12, *P* = 0.219) fails to reach the threshold *P* of 0.05. These results point clearly to the involvement of two genes underlying expression of the virus-neutralizing antibody. Further clarification is gained from examining allele distributions in the peak regions of chromosomes 4 and 7 marked (*) in Figure 2B.

**Figure 2:**
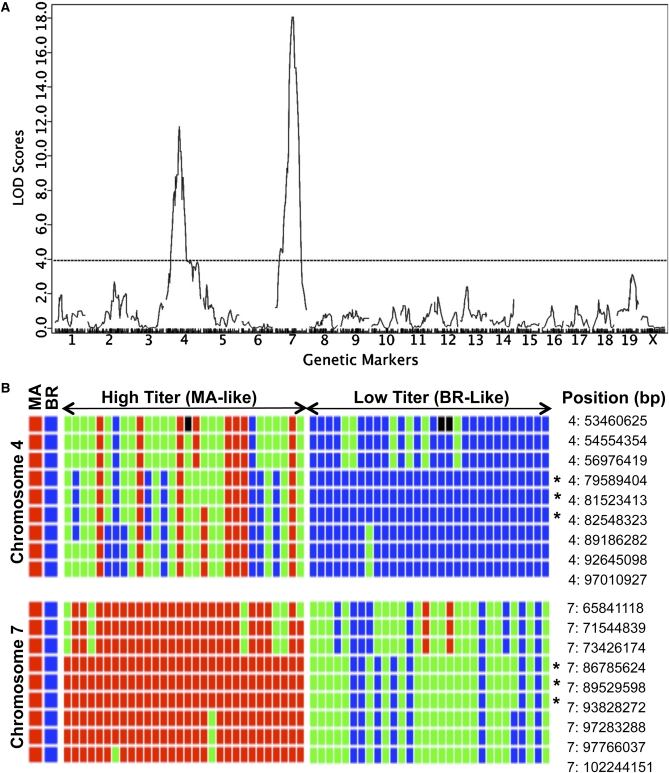
QTL mapping results. (A) DNAs from F2 high-titer and low-titer mice were genotyped by the Genetic Analysis Facility of the Center for Applied Genomics at The Hospital for Sick Children in Toronto, Ontario, with use of the Illumina mouse MD linkage panel. F2 phenotypic and genotypic data (File S1) were imported into along with the genotypic data into J/QTL (http://churchill.jax.org/software/jqtl.shtml), a graphical interface to R/QTL (Broman *et al.* 2003). QTL were identified and plotted with a binary phenotype model with the expectation maximization (EM) algorithm through the “Run One QTL Genome Scan” function. The horizontal dotted line demarcates the genome-wide adjusted *P* = 0.05 threshold estimated with 1,000,000 permutations (Lystig 2003). All reported *P* values are genome-wide adjusted. (B) Genotype plots using the “Display Genotype Plot” function were generated around the SNPs corresponding to the LOD peaks on chromosomes 4 and 7. Each column represents a mouse and each row a SNP. Red, homozygous MA alleles; blue, homozygous BR alleles; green, heterozygous; black, missing allele calls. Asterisks denote the SNPs most strongly linked to the phenotype.

All 30 of the high-titer F2 mice are homozygous for MA alleles throughout the peak region of chromosome 7. These mice show a roughly 1:2:1 segregation of genotypes (MA/MA: MA/BR: BR/BR) in the peak region of chromosome 4. High-titer antibody in these mice is therefore conferred by chromosome 7 independently of the genotype on chromosome 4. Homozygosity of the MA allele on chromosome 7 thus acts in an epistatic manner to the gene on chromosome 4. Of the 30 low-titer F2 mice, all are homozygous for the BR allele on chromosome 4. This homozygous condition is necessary but not sufficient for dictating low titers because none of these mice is homozygous for MA alleles in the peak region of chromosome 7. The ratio of MA/BR to BR/BR mice in the peak region of chromosome 7 is roughly 2:1, reflecting the epistatic effect of the MA gene on chromosome 7 when present in a homozygous condition.

The identities of the genes on chromosomes 4 and 7 and how they may interact remain unknown. To define optimal regions on these chromosomes in which to search for candidate genes, Bayesian 95% credible intervals (Sen and Churchill 2001) were calculated. Based on NCBI Build 37.1 of the mouse genome, the interval on chromosome 4 is 62.5 to 78.2 Mb and on chromosome 7 85.4 to 96.7 Mb. Interestingly, several genes involved in innate immune responses reside in this region of chromosome 7 (Yang *et al.* 2009). Genes involved in innate immune responses to viruses, evolution of natural antibody, or IgM rearrangement, as well as ones possibly linked to radiation resistance, are among the logical ones to pursue.

## Supplementary Material

Supporting Information
