# Supplementary material for: Production of a Natural Antibody to the Mouse Polyoma Virus Is a Multigenic Trait
Source: G3 (Bethesda). 2012 Mar 1;2(3):353–5. doi: 10.1534/g3.111.001701 (PMC3291505; doi:10.1534/g3.111.001701)
Supplement: Supporting Information [file supp_2_3_353__index.html]

Supporting Information 

# Production of a Natural Antibody to the Mouse Polyoma Virus Is a Multigenic Trait

## Supporting Information for Andrews *et al*, 2012

**Files in this Data Supplement:**

- File S1 - Supporting data (.xls, 167 KB)
